# Clinical Response to Anti–Programmed Death 1 After Response and Subsequent Progression on Anti–Programmed Death Ligand 1 Therapy

**DOI:** 10.1200/PO.17.00049

**Published:** 2017-08-15

**Authors:** Emily H. Castellanos, Emily Feld, Monica V. Estrada, Melinda E. Sanders, Pierre P. Massion, Douglas B. Johnson, Justin M. Balko, Leora Horn

**Affiliations:** **All authors**: Vanderbilt University, Nashville TN.

## INTRODUCTION

Immune checkpoint inhibitors are rapidly becoming a cornerstone in the treatment of non–small cell lung cancer (NSCLC). The programmed death 1 (PD-1) receptor and its two ligands, programmed death ligand 1(PD-L1; B7H1) and ligand 2 (PD-L2; B7-DC), negatively regulate T-cell activation, and expression of PD-L1 by tumor cells is an important mechanism of immune evasion.^1-3^ Multiple agents have been developed to disrupt tumor-associated immune evasion by targeting either PD-1 or PD-L1. Nivolumab and pembrolizumab, both anti–PD-1 agents approved for advanced NSCLC progressing after chemotherapy, confer an overall survival benefit and produce response rates in 16% to 23% of unselected patients with NSCLC.^4-6^ Food and Drug Administration approval was also recently extended to pembrolizumab in the first-line setting for patients with metastatic NSCLC expressing PD-L1.^7^ Atezolizumab, an anti–PD-L1 agent, has also demonstrated an overall survival benefit and has been approved for use in the second-line setting.^8^ The growing supply of treatment options has led to new questions of optimal sequencing and choice of therapy. In particular, the utility of serial treatment with immune checkpoint inhibitors has not been established.

Agents targeting PD-L1 block the interaction of PD-L1 expressed on tumor cells and tumor-infiltrating immune cells with PD-1 and B7.1 expressed on T cells. Studies of these agents, including atezolizumab, avelumab, and durvalumab, have demonstrated similar response rates and clinical benefit.^9-14^ Although the effects of anti–PD-L1 are predicted to be similar to anti–PD-1, it has been speculated that the variation in mechanism may lead to distinct antitumor and toxicity profiles compared with the anti–PD-1 agents.^15^ However, these agents have not been directly compared. To our knowledge, this is the first reported case of sequential anti–PD-L1 and anti–PD-1–directed therapy, as well as the first to demonstrate anti–PD-1 activity in an anti–PD-L1 refractory setting.

## CASE REPORT

A 51-year-old former smoker was referred for treatment of progressive metastatic nonsquamous NSCLC. He had been diagnosed approximately 2 years earlier with NSCLC metastatic to bilateral cervical lymph nodes and bilateral adrenal glands. Molecular testing showed that his tumor was *EGFR* and *KRAS* wild type and *ALK* translocation negative. Before this evaluation, he received four lines of therapy, including platinum doublet chemotherapy, single-agent docetaxel, palliative chemoradiation with weekly carboplatin and paclitaxel, and erlotinib.

He was enrolled in a phase I clinical trial of an anti–PD-L1 monoclonal antibody given in 21-day cycles. Computed tomography (CT) imaging after four cycles of therapy (12 weeks) revealed a partial response (PR), with 45% reduction in tumor size. He experienced only mild (grade 1) rash and hypothyroidism. He received a total of 16 cycles of anti–PD-L1 therapy before treatment was stopped per study protocol.

**Fig 1.**
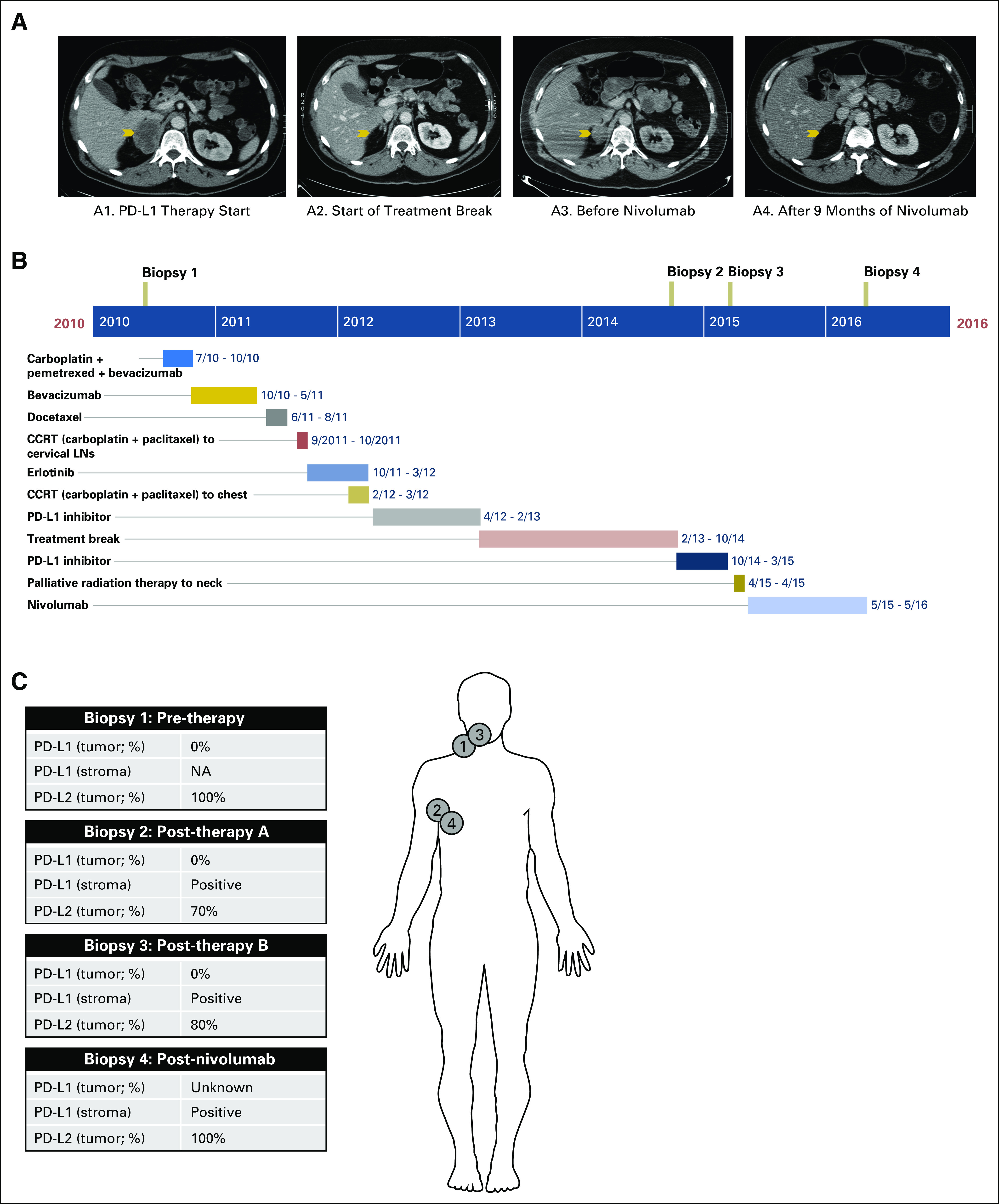
Radiologic and clinical course of disease. (A) Contrasted computed tomography scans of the chest, abdomen, and pelvis show a metastatic lesion within the right adrenal gland (arrow) before initiation of anti–programmed death ligand 1 (PD-L1) therapy (panel A1), which regressed after 16 cycles of treatment with anti–PD-L1 therapy (panel A2). Repeat computed tomography imaging demonstrated progressive disease in the right adrenal gland before initiation of anti–PD-1 therapy (panel A3), with subsequent improvement after 9 months of anti–PD-1 therapy (panel A4). (B) Timeline of therapies. (C) Sites of biopsies and summary of PD-L1/programmed death ligand 2 (PD-L2) staining. CCRT, concurrent radiotherapy; LNs, lymph nodes; NA, not available.

The patient was followed with serial cross-sectional imaging and demonstrated continued PR on imaging for 16 months after therapy completion. He then developed pain and swelling in his neck. CT imaging revealed enlarging cervical and right axillary lymphadenopathy and growth of two previously noted nodules in his right adrenal gland. He restarted anti–PD-L1 therapy per study protocol, with CT imaging after 8 weeks showing stable disease. After seven cycles of treatment, he was noted to have progression of disease in his right axilla and a new retropharyngeal (RP) mass. Biopsy of the RP mass showed poorly differentiated carcinoma consistent with recurrent NSCLC and similar to his initial biopsy. He was treated with palliative radiation therapy (30 Gy in 10 fractions) to both his right axilla and RP space. Two weeks after radiation therapy, repeat imaging demonstrated stability of the RP mass, decrease in the size of the right axillary lymph node, and enlarging adrenal masses. He was given nivolumab 3 mg/kg every 2 weeks. Imaging of the face after 6 weeks showed a PR in the RP space, and body imaging after 8 weeks of treatment showed a PR in both the right axilla and right adrenal gland (Fig 1A). He tolerated treatment well, with no immune-related adverse events for 24 cycles, after which he was noted to have progression limited to his right axilla, with a core biopsy confirming NSCLC. This sample was sent for genomic testing through FoundationONE. Given that this represented isolated progressive disease, he continued receiving nivolumab and underwent a right axillary lymph node dissection for local disease control. Unfortunately, he died several days after this procedure at an outside facility; the cause of death was unclear.

### Statement of Informed Consent

All human investigations were performed after approval by a local human investigations committee and in accord with an assurance filed with and approved by the Department of Health and Human Services, and all data were anonymized to protect the identities of participants involved in the research. Informed consent from the patient for such research was obtained.

### Longitudinal Assessment of PD-L1 and PD-L2 Expression

**Fig 2.**
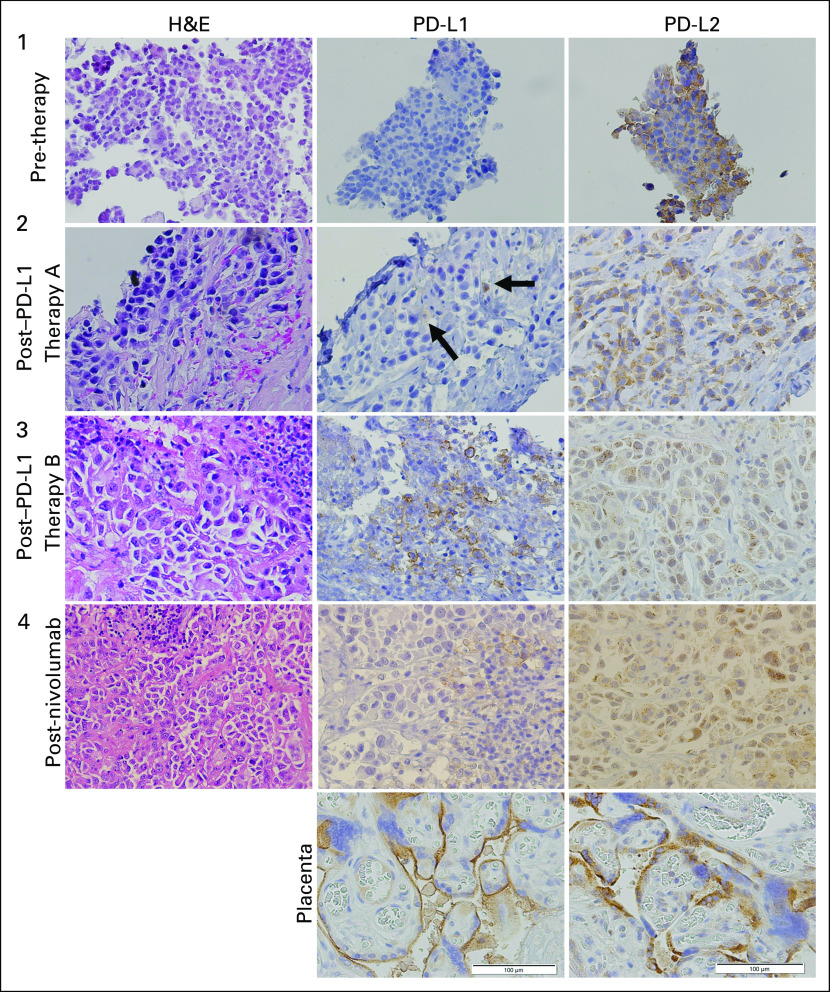
Histology of longitudinal specimens. Representative immunohistochemistry images at pretherapy (biopsy 1), and post-therapy (before second anti–programmed death ligand 1 [PD-L1] course; post-PD-L1 therapy A; biopsy 2), at progression/resistance to PD-L1 therapy (before response to nivolumab; post–PD-L1 therapy B; biopsy 3), and at nivolumab resistance (postnivolumab; biopsy 4). H&E, hematoxylin and eosin; PD-L2, programmed death ligand 2.

Biopsy samples were obtained at the time of diagnosis (biopsy 1), before repeat treatment with anti–PD-L1 (biopsy 2), before treatment with anti-PD-1 (biopsy 3), and after progression anti–PD-1 therapy (biopsy 4; Fig 1B). At the time of diagnosis, the tumor sample was PD-L1 negative, but strongly PD-L2 positive (100%). However, tissue for this first sample was extremely limited, and possible PD-L1 positivity could not be ruled out because of the size of the tissue specimen, particularly because subsequent specimens from the patient demonstrated a highly heterogeneous pattern of PD-L1 expression with rare positive stromal cells, whereas PD-L2 staining was robust throughout the tumor. After his initial disease progression while receiving anti–PD-L1 therapy, repeat biopsy demonstrated no tumor PD-L1 staining (with limited stromal PD-L1 positivity), and PD-L2 remained strongly positive (70%; Fig 1C). Similar results were demonstrated in biopsy 3 before starting PD-1 therapy and biopsy 4 after progression on this final line of treatment, with strong PD-L2 expression in tumor, moderate PD-L1 staining in stroma, and absence of PD-L1 expression in tumor. Representative immunohistochemistry samples from each time point are shown in Figure 2.

### Targeted Next-Generation Sequencing

Genomic testing using a hybrid capture-based next-generation sequencing platform assessing exons from 315 genes (FoundationONE) of the patient’s tumor at the time of progression while taking nivolumab (biopsy 4) identified seven potentially functional alterations, including *RICTOR* amplification, *ARID1A* T2030fs*3, *ARID2* Q904*, *KDM5C* E656*, *SLIT2* G498*, *TET2* E1851*, and *TP53* R280I. Thirty-three variants of unknown significance were identified, and a total mutation burden of 53 mutations/megabase was estimated, placing this patient in the top 7% of all tumor specimens tested by this method.^16^ Thus, the response observed in this high mutation-load patient was consistent with clinical observations.^17^ No alterations were identified in *JAK1*, *JAK2*, *CD274, PDCD1LG2, PTEN*, *MYC*, or *CTNNB1* in this patient, which were previously reported mechanisms of T-cell exclusion and/or resistance to anti–PD-1 therapy.^18-21^ Next-generation sequencing data were only available on the final biopsy specimen; thus, changes in mutation burden over time cannot be assessed.

### Gene Expression Analysis

**Fig 3.**
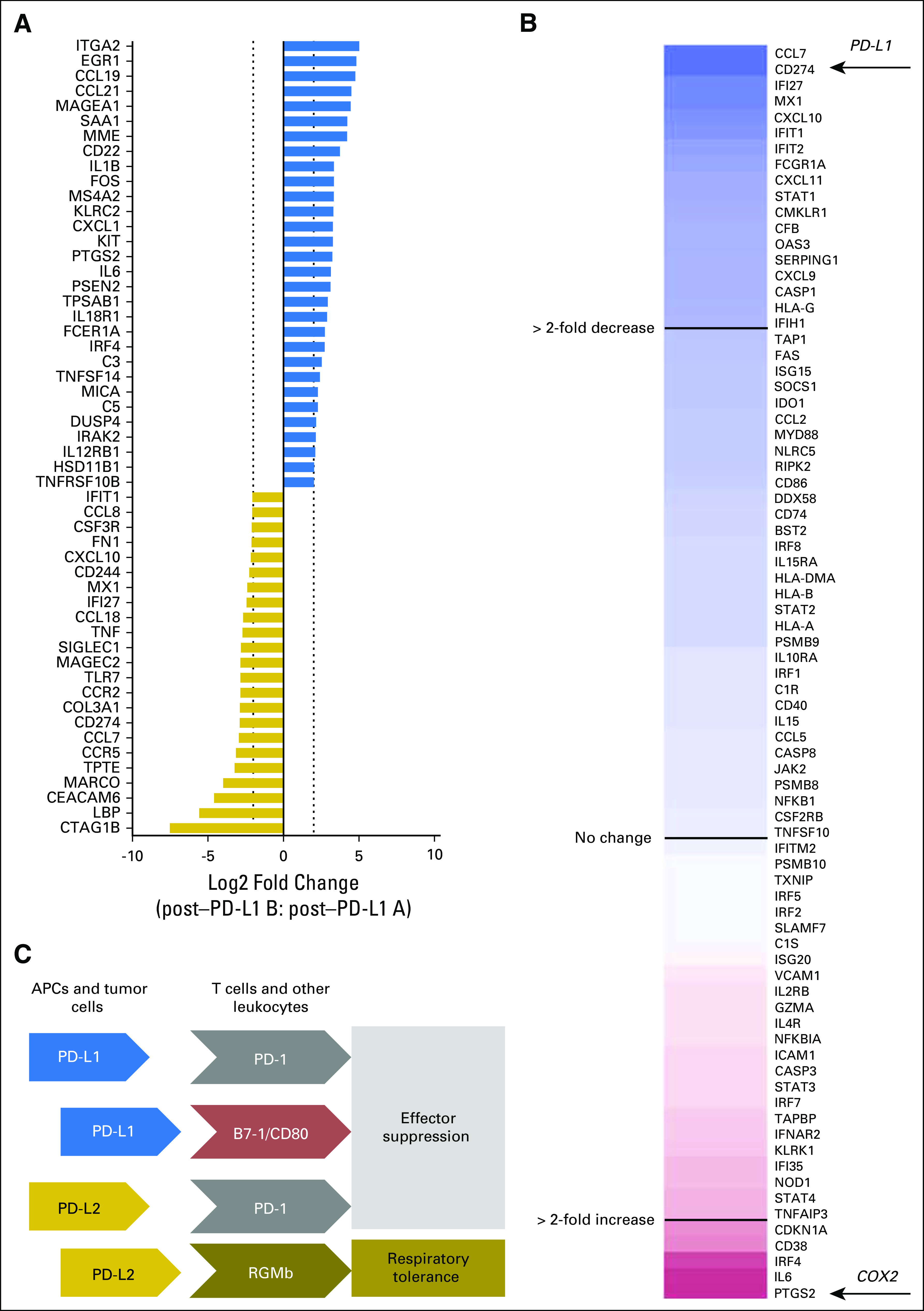
Molecular analysis of longitudinal tumor specimens. (A) RNA collected from archival tissue blocks were analyzed by nanoString PanCancer Immune Profiling (730 immune-related genes). Normalized data were compared between the post-therapy A (preresistance) and post-therapy B (postresistance) specimens for log2 fold change. Fold change for genes altered by > 2 log2 units (four-fold change) are shown. (B) Genes involved in the interferon-gamma signaling reactome (www.reactome.org; accession: M965) were selected from the PanCancer Immune Profiling gene set and plotted by fold change. This analysis demonstrated a general decrease in interferon-gamma signaling within the tumor microenvironment at disease progression on anti–programmed death ligand 1 (PD-L1). (C) Simplified schematic of known PD-L1/programmed death ligand 2 (PD-L2) interactions. PD-L1 interacts with programmed death 1 (PD-1) and B7-1 (CD80) to promote immune effector cell suppression, whereas PD-L2 can bind PD-1 and RGMb, promoting effector suppression and respiratory tolerance in lung-resident macrophages, respectively.^22,23^ APCs, antigen-presenting cells; COX2, cyclooxygenase-2.

Sufficient tissue for expression analysis (nanoString PanCancer Immune Profiling Panel) was available for two of the four biopsy time points: post-therapy A, which was sampled before beginning the second treatment course of anti–PD-L1, and post-therapy B, sampled at disease progression on anti–PD-L1, before radiation therapy followed by nivolumab. Comparison of the expression of immune genes between these two samples demonstrated substantial downregulation of *CD274* (PD-L1) mRNA and upregulation of several immunosuppressive genes, including *IL6* and *PTGS2* (cyclooxygenase-2; Fig 3A). Interferon-gamma–responsive genes demonstrated a global downregulation in the resistant sample, consistent with the predicted effects of a decrease in T-cell activity in the tumor microenvironment (Fig 3B).

## DISCUSSION

Although immunotherapies are being rapidly adopted into widespread use for the treatment of NSCLC, reasons for progression on therapies targeting the PD-1/PD-L1 axis and subsequent activity of alternate immune therapies after progression are unknown. This case provides clinical evidence suggesting that these agents may have nonoverlapping mechanisms of response. It could be hypothesized that distinct immune checkpoints (eg, PD-L1 *v* PD-L2) could divergently mediate therapeutic resistance to checkpoint inhibitors and support preclinical studies testing this hypothesis.

**Fig A1.**
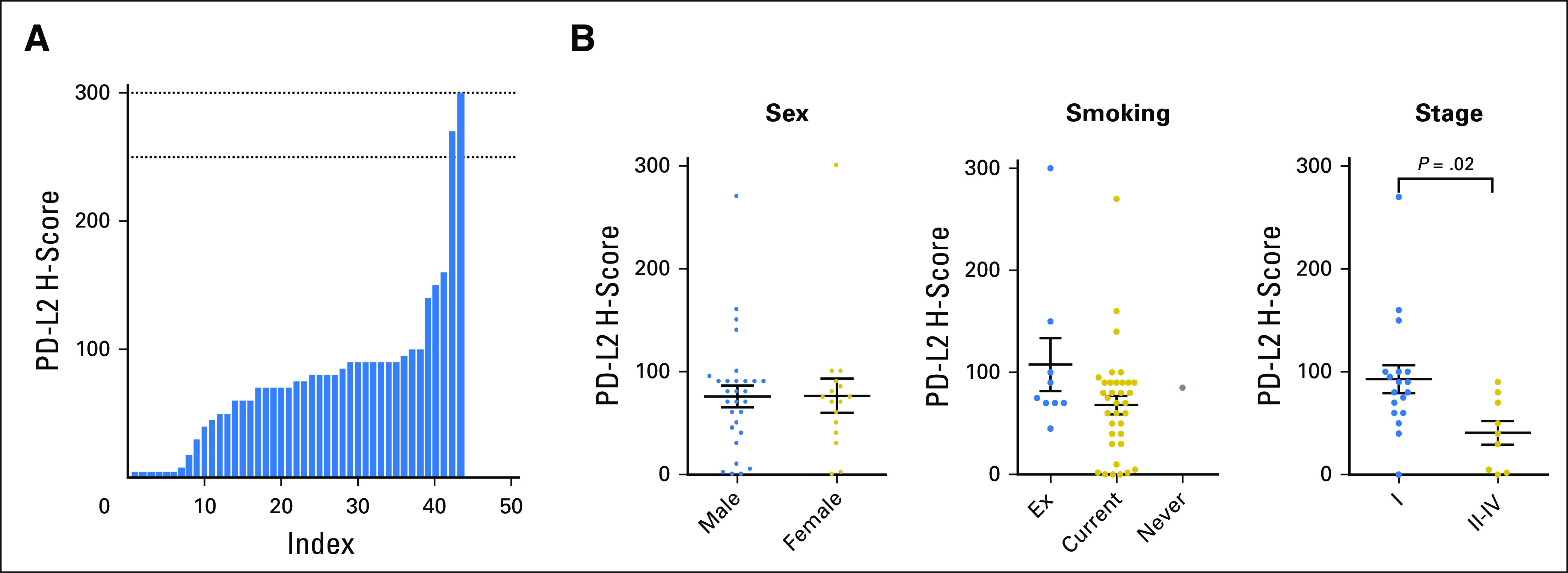
Programmed death ligand 2 (PD-L2) expression across 43 patients with non–small cell lung cancer (NSCLC). (A) A tissue microarray series of 43 patients with NSCLC was stained for PD-L2 expression and analyzed by histoscore (H-score; % of cells staining positive × intensity [0-3+]). Dotted lines between 250 and 300 show the H-score range for PD-L2, in which the longitudinal specimens from the patient fell. (B) PD-L2 expression and its association with sex, smoking, and stage. *P* value represents the result of a two-tailed Student *t* test.

This patient’s tumor was PD-L1 negative at diagnosis, with some positivity in the stroma, on the basis of our limited sample, but strongly PD-L2 positive. This finding suggests that the significance of PD-L2 positivity in response to PD-L1 or PD-1 targeted therapies may be a useful subject of study. PD-L2 is primarily expressed on antigen-presenting cells, including macrophages and dendritic cells.^1^ This is in contrast to PD-L1, which is more ubiquitous and present in peripheral tissues on resting T cells, B cells, dendritic cells, macrophages, vascular endothelial cells, and pancreatic islet cells.^24^ PD-1 binds both PD-L1 and PD-L2, but interestingly, the relative affinity of PD-L2 to PD-1 is approximately two to six times greater than that of PD-L1.^25^ The physiologic and clinical significance of this remains unclear. Examination of PD-L2 expression across multiple tumor types found that although PD-L2 is often coexpressed with PD-L1, isolated PD-L2 expression does occur.^26^ In lung cancer, several reports have evaluated expression of PD-L2 in larger cohorts and reported the prevalence of PD-L2 in 23.9% (squamous cell carcinoma) and 47% (KRAS-mutant NSCLC) of patients, although these studies used different clones for detection.^27,28^ Using our own methods for detection of PD-L2 in a tissue microarray cohort of 43 patients with NSCLC, we found that this patient was consistently among the top 5% of PD-L2 expressers. PD-L2 expression seemed to be associated with earlier stage of disease (stage I *v* all others), but not with gender or smoking status (Appendix Fig A1).

During the second treatment with anti–PD-L1, which was accompanied by short-term stable disease and subsequent progression, we observed maintenance of high PD-L2 expression and downregulation of PD-L1 mRNA expression accompanied by simultaneous decrease of interferon-gamma response signatures. PD-L1 staining in samples flanking this treatment yielded rare populations of positive stromal cells. We also observed upregulation of immunosuppressive genes, such as cyclooxygenase-2 (recently shown to be important in mediating anti–PD-1 responsiveness^29^) and interleukin-6, which was shown to decrease after initial treatment with PD-L1 targeted therapy.^30^ Thus, the changes in gene expression within the tumor microenvironment in this patient were consistent with previous studies.

Importantly, there were multiple intervening therapies in this patient during the approximately 20-month period after the initial pretreatment biopsy, limiting the inferences that can be made during the initial therapy with anti–PD-L1. There were no additional intervening therapies between the flanking biopsies of the second trial of anti–PD-L1.

Abscopal effects of radiation with immunotherapy have been reported in several tumor types, particularly in conjunction with anti–cytotoxic T-cell lymphocyte-4 agents.^31-33^ Although the mechanism of the abscopal effect is not entirely understood, upregulation of PD-L1 and PD-L2 has been demonstrated in tumor models after radiation therapy.^34,35^ None of our patient’s biopsies demonstrated upregulation of PD-L1 despite his prior treatments with palliative radiation; however, he did receive an additional course of palliative radiation after his third biopsy and preceding his anti–PD-1 therapy, and a role for radiation in sensitizing his tumor to anti-PD-1 therapy cannot be ruled out.

Although anecdotal, this case report demonstrates an important clinical finding: patients who become resistant to anti–PD-L1 may still benefit from PD-1 targeted therapies, possibly due to a switch from dependency on PD-L1 to PD-L2 for maintenance of immunosuppression. However, the presence of consistently high PD-L2 staining argues against a direct mechanism of PD-L2–mediated de novo resistance to anti–PD-L1 therapy. Moreover, *PD-L2* mRNA did not seem to be upregulated or changed during acquisition of resistance to anti–PD-L1 therapy. However, the ratio of *PD-L2* to *PD-L1* mRNA increased substantially from 3.6-fold to 14.6-fold during this period and may be a useful metric to test experimentally in the future for association with resistance to anti–PD-L1 treatment. Although more investigation is needed in the context of preclinical studies and clinical trials, this report supports investigations of sequencing anti–PD-L1 and anti–PD-1 therapies to elucidate mechanisms of cross-resistance and derive maximal patient benefit from these agents. Nonetheless, we strongly feel that patients should not be sequenced in this manner outside of a clinical trial in the absence of supportive systematically collected data on the utility of sequencing immunotherapies in NSCLC.

## References

[1] Keir ME Butte MJ Freeman GJ et al: PD-1 and its ligands in tolerance and immunity. Annu Rev Immunol 26:677-704, 2008 18173375 10.1146/annurev.immunol.26.021607.090331 PMC10637733

[2] Mu CY Huang JA Chen Y et al: High expression of PD-L1 in lung cancer may contribute to poor prognosis and tumor cells immune escape through suppressing tumor infiltrating dendritic cells maturation. Med Oncol 28:682-688, 2011 20373055 10.1007/s12032-010-9515-2

[3] Francisco LM Salinas VH Brown KE et al: PD-L1 regulates the development, maintenance, and function of induced regulatory T cells. J Exp Med 206:3015-3029, 2009 20008522 10.1084/jem.20090847 PMC2806460

[4] Garon EB Rizvi NA Hui R et al: Pembrolizumab for the treatment of non–small-cell lung cancer. N Engl J Med 372:2018-2028, 2015 25891174 10.1056/NEJMoa1501824

[5] Borghaei H Paz-Ares L Horn L et al: Nivolumab versus docetaxel in advanced nonsquamous non–small-cell lung cancer. N Engl J Med 373:1627-1639, 2015 26412456 10.1056/NEJMoa1507643 PMC5705936

[6] Brahmer J Reckamp KL Baas P et al: Nivolumab versus docetaxel in advanced squamous-cell non–small-cell lung cancer. N Engl J Med 373:123-135, 2015 26028407 10.1056/NEJMoa1504627 PMC4681400

[7] Reck M Rodríguez-Abreu D Robinson AG et al: Pembrolizumab versus chemotherapy for PD-L1-positive non–small-cell lung cancer. N Engl J Med 375:1823-1833, 2016 27718847 10.1056/NEJMoa1606774

[8] Rittmeyer A Barlesi F Waterkamp D et al: Atezolizumab versus docetaxel in patients with previously treated non–small-cell lung cancer (OAK): A phase 3, open-label, multicentre randomised controlled trial. Lancet 389:255-265, 2017 27979383 10.1016/S0140-6736(16)32517-X PMC6886121

[9] Brahmer JR Tykodi SS Chow LQ et al: Safety and activity of anti-PD-L1 antibody in patients with advanced cancer. N Engl J Med 366:2455-2465, 2012 22658128 10.1056/NEJMoa1200694 PMC3563263

[10] Brahmer JR, Rizvi NA, Luzky J, et al: Clinical activity and biomarkers of MEDI4736, an anti-PD-L1 antibody, in patients with NSCLC. J Clin Oncol 32, 2014 (suppl; abstr 8021)

[11] Rizvi N, Brahmer J, Ignatius S, et al: Safety and clinical activity of MEDI4736, an anti-programmed cell death-ligand 1 (PD-L1) antibody, in patients with non–small-cell lung cancer (NSCLC). J Clin Oncol 33, 2015 (suppl; abstr 8032)

[12] Spira AI, Park K, Mazières J, et al: Efficacy, safety and predictive biomarker results from a randomized phase II study comparing MPDL3280A vs docetaxel in 2L/3L NSCLC (POPLAR). J Clin Oncol 33, 2015 (suppl; abstr 8010 )

[13] Spigel DR, Chaft JE, Gettinger SN, et al: Clinical activity and safety from a phase II study (FIR) of MPDL3280A (anti-PDL1) in PD-L1–selected patients with non–small-cell lung cancer (NSCLC). J Clin Oncol 33, 2015 (suppl; abstr 8028)

[14] Kelly K, Patel MR, Infante JR, et al: Avelumab (MSB0010718C), an anti-PD-L1 antibody, in patients with metastatic or locally advanced solid tumors: Assessment of safety and tolerability in a phase I, open-label expansion study. J Clin Oncol 32, 2015 (suppl; abstr 3044)

[15] Johnson DB Rioth MJ Horn L: Immune checkpoint inhibitors in NSCLC. Curr Treat Options Oncol 15:658-669, 2014 25096781 10.1007/s11864-014-0305-5 PMC4216599

[16] Frampton GM, Fabrizio D, Chalmers ZR, et al: Assessment of tumor mutation burden from >60,000 clinical cancer patients using comprehensive genomic profiling. J Clin Oncol 34, 2016 (suppl; abstr 11558)

[17] Johnson DB, Frampton GM, Rioth MJ, et al: Hybrid capture-based next-generation sequencing (HC NGS) in melanoma to identify markers of response to anti-PD-1/PD-L1. J Clin Oncol 33, 2016 (suppl; abstr 105)

[18] Zaretsky JM, Garcia-Diaz A, Shin DS, et al: Mutations associated with acquired resistance to PD-1 blockade in melanoma. N Engl J Med 375:819-829, 2016 10.1056/NEJMoa1604958 PMC5007206 27433843

[19] Peng W Chen JQ Liu C et al: Loss of PTEN promotes resistance to T cell-mediated immunotherapy. Cancer Discov 6:202-216, 2016 26645196 10.1158/2159-8290.CD-15-0283 PMC4744499

[20] Spranger S Bao R Gajewski TF: Melanoma-intrinsic β-catenin signalling prevents anti-tumour immunity. Nature 523:231-235, 2015 25970248 10.1038/nature14404

[21] Casey SC Tong L Li Y et al: MYC regulates the antitumor immune response through CD47 and PD-L1. Science 352:227-231, 2016 26966191 10.1126/science.aac9935 PMC4940030

[22] Xiao Y Yu S Zhu B et al: RGMb is a novel binding partner for PD-L2 and its engagement with PD-L2 promotes respiratory tolerance. J Exp Med 211:943-959, 2014 24752301 10.1084/jem.20130790 PMC4010901

[23] Chen L Han X: Anti-PD-1/PD-L1 therapy of human cancer: Past, present, and future. J Clin Invest 125:3384-3391, 2015 26325035 10.1172/JCI80011 PMC4588282

[24] He J Hu Y Hu M et al: Development of PD-1/PD-L1 Pathway in tumor immune microenvironment and treatment for non–small-cell lung cancer. Sci Rep 5:13110, 2015 26279307 10.1038/srep13110 PMC4538573

[25] Youngnak P Kozono Y Kozono H et al: Differential binding properties of B7-H1 and B7-DC to programmed death-1. Biochem Biophys Res Commun 307:672-677, 2003 12893276 10.1016/s0006-291x(03)01257-9

[26] Yearley J, Gibson C, Yu N, et al: PD-L2 expression in human tumors: Relevance to anti-PD-1 therapy in cancer. Presented at European Society for Medical Oncology Annual Meeting, Vienna, Austria, September 25-29, 2015

[27] Calles A Liao X Sholl LM et al: Expression of PD-1 and its ligands, PD-L1 and PD-L2, in smokers and never smokers with KRAS-mutant lung cancer. J Thorac Oncol 10:1726-1735, 2015 26473645 10.1097/JTO.0000000000000687

[28] Kim MY Koh J Kim S et al: Clinicopathological analysis of PD-L1 and PD-L2 expression in pulmonary squamous cell carcinoma: Comparison with tumor-infiltrating T cells and the status of oncogenic drivers. Lung Cancer 88:24-33, 2015 25662388 10.1016/j.lungcan.2015.01.016

[29] Zelenay S van der Veen AG Böttcher JP et al: Cyclooxygenase-dependent tumor growth through evasion of immunity. Cell 162:1257-1270, 2015 26343581 10.1016/j.cell.2015.08.015 PMC4597191

[30] Herbst RS Soria JC Kowanetz M et al: Predictive correlates of response to the anti-PD-L1 antibody MPDL3280A in cancer patients. Nature 515:563-567, 2014 25428504 10.1038/nature14011 PMC4836193

[31] Golden EB Demaria S Schiff PB et al: An abscopal response to radiation and ipilimumab in a patient with metastatic non–small cell lung cancer. Cancer Immunol Res 1:365-372, 2013 24563870 10.1158/2326-6066.CIR-13-0115 PMC3930458

[32] Hiniker SM Chen DS Knox SJ: Abscopal effect in a patient with melanoma. N Engl J Med 366:2035, author reply 2035-2036, 2012 22621637 10.1056/NEJMc1203984

[33] Postow MA Callahan MK Barker CA et al: Immunologic correlates of the abscopal effect in a patient with melanoma. N Engl J Med 366:925-931, 2012 22397654 10.1056/NEJMoa1112824 PMC3345206

[34] Deng L Liang H Burnette B et al: Irradiation and anti-PD-L1 treatment synergistically promote antitumor immunity in mice. J Clin Invest 124:687-695, 2014 24382348 10.1172/JCI67313 PMC3904601

[35] Diamond J, Pilones K, Aryankalayil J, et al: Radiotherapy induces responsiveness of a resistant mammary carcinoma to PD-1 blockade. J Immunother Cancer 2:P159, 2014 (suppl 3)

